# Clinicopathological factors associated with recurrence in patients undergoing resection of pancreatic solid pseudopapillary neoplasm

**DOI:** 10.1007/s12672-021-00451-4

**Published:** 2021-11-22

**Authors:** Oscar Paredes, Kori Paredes, Yoshikuni Kawaguchi, Carlos Luque-Vasquez, Iván Chavez, Juan Celis, Eduardo Payet, Eloy Ruiz, Francisco Berrospi

**Affiliations:** 1 Department of Abdominal Surgery, National Institute of Neoplastic Diseases INEN, Lima, Peru; 2 Hepato-Biliary-Pancreatic Surgery Division, Department of Surgery, Graduate School of Medicine, The University of Tokyo, Tokyo, Japan; 3 Montesquieu Street 277, Lima, Perú

**Keywords:** Pancreas, Pseudopapillary neoplasm (SPN), Recurrence

## Abstract

**Purpose:**

Solid pseudopapillary neoplasm (SPN) is an uncommon pathology with a low-grade malignancy. Surgery is the milestone treatment. Nevertheless, despite appropriate management, some patients present recurrence. Risk factors associated with recurrence are unclear. The objective was to identify the clinicopathological factors associated with recurrence in patients with SPN treated with pancreatic resection.

**Methods:**

Medical records of patients treated with pancreatic resection during 2006–2020 were evaluated. Patients with histological diagnosis of SPN were included. Survival analysis was performed to identify the clinicopathological factors related to recurrence.

**Results:**

Seventy-four patients were diagnosed with SPN; 70 (94.6%) patients were female, and the median age was 20 years old. The median tumor diameter was 7.9 cm. Multivisceral resection was performed in 9 (12.2%) patients. Four (5.4%) patients presented lymph node metastasis.R0 resection was achieved in all cases. Six (8%) patients presented recurrence and the liver was the most frequent recurrence site (n = 5).After a median follow-up of 40.2 months, 9 (12%) patients died. Five (6.8%) patients died of disease progression. The 1–3- and 5-year overall survival (OS) was 97.1%, 90.2% and 79.9%, respectively. The 1–3-and-5-year recurrence-free survival (RFS) was 98.4%, 89.9% and 87%, respectively. In the univariate Cox-regression analysis, age ≥ 28 years(HR = 8.61, 95% CI 1.1–73.8),tumor diameter ≥ 10 cm(HR = 9.3, 95% CI 1.12–79.6),invasion of adjacent organs (HR = 7.45, 95% CI 1.5–36.9), lymph node metastasis (pN +) (HR = 16.8, 95% CI 2.96–94.9) and, AJCC Stage III (HR = 10.1, 95% CI 1.2–90.9) were identified as predictors for recurrence.

**Conclusions:**

SPN is more frequently diagnosed in young women with a good overall prognosis after an R0 surgical resection even with disease recurrence. Age ≥ 28 years, larger tumors ≥ 10 cm, invasion of adjacent organs, lymph node metastasis(pN +) and, AJCC Stage III were predictors factors of recurrence in resected SPN.

**Supplementary Information:**

The online version contains supplementary material available at 10.1007/s12672-021-00451-4.

## Background

Solid pseudopapillary neoplasm (SPN) of the pancreas, first described by Frantz in 1959 [1], is a relatively rare disease that represents 1–3% of all exocrine pancreatic tumors [2, 3]. SPN is mostly diagnosed in young women (mean age; 28 years old) and presents a female to male ratio of 9.8/1[4]. The tail of the pancreas is the most frequent localization and, most patients are symptomatic [5]. The pathogenesis of SPN is unclear, mutation of the exon 3 of the beta-catenin gene, FLI-1, DOG1, BCL9L expression, and hormone sensitivity had been studied but conclusive evidence is limited [6–10]. In the literature it could be defined as a solid-papillary tumor, solid-cystic acinar tumor, Hamoudi or Frantz tumor, papillary-cystic tumor [11, 12]; and, in the WHO classification 2010 [13], this tumor was renamed as SPN and defined as a low-grade malignant neoplasm.

Radical surgery is the principal treatment [14], and; 9–13% of resected SPN presents malignant behavior including local spread and remote organ metastasis [5, 15–18]. Studies reported the following factors associated with prognosis: lymphovascular invasion, perineural invasion, distant metastasis, the involvement of surrounding tissue. However, the results remain controversial because of the small number of studies and patients [4, 11]. Although some SPN shows malignant comportment, the prognosis of SPN after surgery is generally good with a 5-year overall survival > 95% [19]. As such, it is important to identify patients with SPN who have a poor prognosis for tailoring surveillance after surgery. Our study aims to evaluate clinicopathological factors associated with recurrence in patients diagnosed with SPN.

## Methods

### Study population

Patients who underwent resection of pancreas benign and malignant primary tumors with curative intent at the National Institute of Neoplastic Diseases INEN from January 2006 through January 2020 were identified from a prospectively compiled database. Patients with pathological diagnoses of SPN were included in the study. SPN was diagnosed based on the current WHO classification [20]. The study was approved by the institutional review board at the National Institute of Neoplastic Diseases INEN.

### Preoperative assessment

Preoperative assessment of the thorax, abdomen, and pelvis was performed using computed tomography with a contrast agent. Magnetic resonance imaging was performed if necessary. A completed blood count, coagulation profile, renal and hepatic function test, tumor markers including Carcinoembryonic antigen (CEA) (< 5 ng/mL) and Carbohydrate antigen 19-9 (CA19-9) (< 37 U/mL) were examined. Additionally, nutritional, psychological, and cardiological evaluations were performed. We did not perform a preoperative tumor biopsy.

### Surgical procedure

**Fig. 1:**
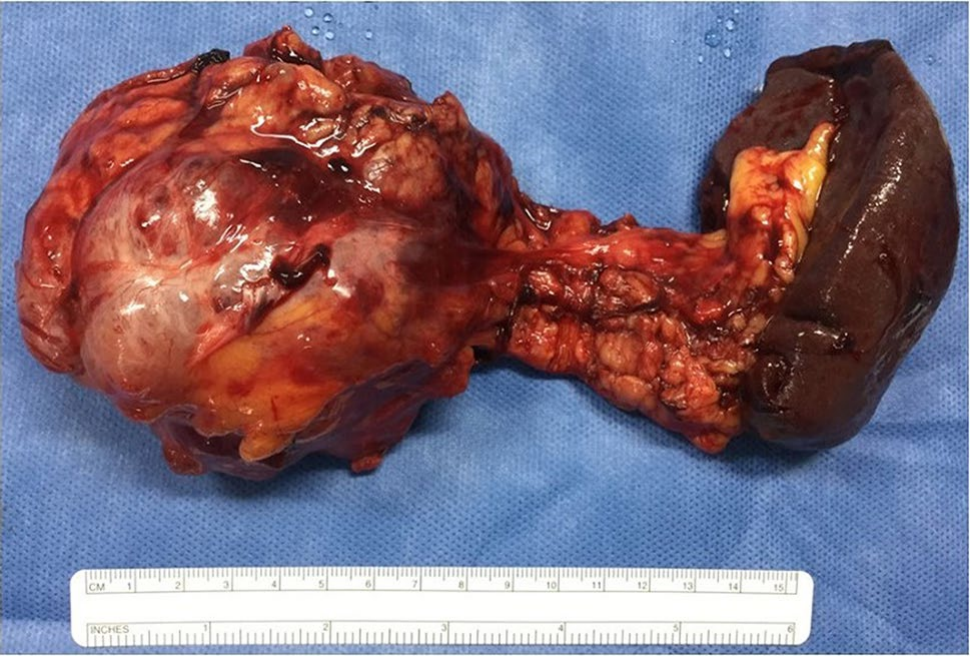
Solid pseudopapillary neoplasm. Surgical specimen (distal pancreatectomy): large round well-defined tumor located in the pancreas tail

The surgical procedure was selected based on the tumor localization. Conventional pancreaticoduodenectomy (PD) or pylorus-preserving PD was performed for tumors located in the pancreas head. Two techniques were performed for pancreato-enteric anastomosis (duct-to-mucosa and modified telescopic), depending on the diameter of the pancreatic duct and the pancreas texture. Conventional distal pancreatectomy (with resection of the spleen) or splenic vessel-preserving distal pancreatectomy were performed for tumors located in the pancreas tail (Fig. 1) [21, 22]. For the tumors located at the neck and proximal body of the pancreas, a central pancreatectomy was performed. Multivisceral resection was performed in cases in which the tumor invaded adjacent organs.

### Postoperative management

Postoperative complications were classified according to the Clavien-Dindo classification [23]. The pancreatic fistula (PF) was defined based on the International Study Group of Pancreatic Fistula (ISGPF) [24]. Namely, PF was diagnosed with amylase level in fluid from the abdominal drainage tube > 3 times the upper limit of normal serum amylase level from the third postoperative day. According to the 2016 update of the ISGPF, pancreatic fistula grade A was redefined as a biochemical leak because of the absence of clinical relevance [25]. Postpancreatectomy hemorrhage (PPH) was defined according to the International Study Group of Pancreatic Surgery [26]. Postoperative morbidity and mortality were defined as complications and mortality which occurred within 90 days after surgery.

### Follow-up

In our institution, patients treated with pancreatic resection were followed up after 2 weeks, 1 month, and later every 3 months for 1 year. In the second year, patients were followed up every 6 months and later annually from the third to fifth year. After the fifth year, patients continue their clinical evaluations in their locality and annually in our institution. In case of suspected disease recurrence, patients were sent to our center immediately. Each medical evaluation included: clinical examination, tumor markers (CEA, CA 19-9), chest X-ray, and abdominopelvic ultrasound. Pelvic computed tomography (CT) or Magnetic resonance image (MRI) was performed every year for 5 years.

### Statistical analysis

Categorical variables were expressed as numbers and percentages and compared using Fisher’s exact test or χ^2^ test as appropriate. Continuous variables were expressed as median values with interquartile range (IQR) and compared using the non-parametric Mann–Whitney test. An analysis of the receiver operating characteristic curve (ROC curve) was used to obtain the optimal cut-off values for age and tumor diameter. Optimal cut-off values were used for the dichotomization of the variables in the univariate analysis. The optimal cut-off point for age was ≥ 28 years and for tumor diameter was ≥ 10 cm. Each AJCC stage and its relationship with the recurrence were evaluated separately; AJCC Stage III showed a statistically significant relationship with recurrence and was used for the variable dichotomization. Survival curves including overall survival (OS), and recurrence-free survival (RFS) were estimated using the Kaplan–Meier method. Deaths without recurrence were censored for the RFS analysis. A Cox proportional hazards model analysis was performed to identify factors associated with recurrence. The statistical analysis was performed with IBM SPSS version 22.0. Statistical significance was defined as *p* ≤ 0.05.

### Ethics approval

This study was performed in line with the principles of the Declaration of Helsinki. Approval was granted by the Ethics Committee of the National Institute of Neoplastic Diseases INEN, Lima, Peru. The authors declare that this article does not contain personal information that allows identifying patients enrolled.

## Results

### Clinical characteristics

From January 2006 to January 2020, a total of 589 patients underwent pancreatic resection. Of these, 74 (12.6%) patients including 70 (94.6%) female patient and 4 (5.4%) male patients were diagnosed as SPN. Demographic and clinicopathologic characteristics were shown in Table 1. The median (IQR) age was 20 (18.2) years, and 49 (67%) patients were < 28 years old. The principal symptom was abdominal pain in 62 (83.8%) patients followed by nausea and vomiting (n = 15; 20.3%) and palpable abdominal mass (n = 8; 10.8%). The median (IQR) tumor diameter was 7.9 (5.8) cm, and 50 (68%) patients presented tumors < 10 cm in diameter. Tumor location is as follows: the pancreas head (n = 32; 43.2%), the pancreas tail (n = 26; 35.2%) and the pancreas body (n = 16; 21.6%). The median (IQR) CEA and CA 19-9 levels were 1.1 (0.9) ng/mL and 6.9 (9.8) U/mL, respectively. No patients presented metastatic disease at presentation.

**Table 1:** Clinical characteristics in 74 patients with solid pseudopapillary neoplasm who underwent surgery

	Total
	n^a^ = 74
Patient factors	
Age^b^, median (IQR)^c^, years	20 (18.2)
< 28 n, (%)	49 (67)
≥ 28 n, (%)	25 (33)
Sex, n (%)	
Female	70 (94.6)
Male	4 (5.4)
Symptoms	
Abdominal pain, n (%)	62 (83.8)
With nausea and vomiting, n (%)	15 (20.3)
With jaundice, n (%)	3 (4)
With weight loss, n (%)	4 (5.4)
Palpable abdominal mass, n (%)	8 (10.8)
None, n (%)	4 (5.4)
CEA^d^ level, median (IQR), *n*g/mL	1.1 (0.9)
CA 19-9^e^ level, median (IQR), U/mL	6.9 (9.8)
Tumor diameter, median (IQR), cm	7.9 (5.8)
< 10 n, (%)	50 (68)
≥ 10 n, (%)	24 (32)
Tumor location	
Head, n (%)	32 (43.2)
Body, n (%)	16 (21.6)
Tail, n (%)	26 (35.2)

^a^n: number of patients

^b^At the date of surgery

^c^IQR: interquartile range

^d^CEA: carcino embryonic antigen

^e^CA 19-9: carbohydrate antigen 19-9

### Surgical and pathological characteristics

Surgical and histopathological outcomes were shown in Table 2. Of the 74 patients, 33 (44.6%) underwent distal pancreatectomy, 32 (43.2%) underwent pancreaticoduodenectomy and, 9 (12.2%) underwent central pancreatectomy. The median (IQR) operative time was 300 (168) minutes and, the median (IQR) estimated blood loss was 275 (275) mL. Resection of other organs was performed in 9 (12.2%) patients as follows: right hemicolectomy (n = 4), transverse colectomy (n = 2), segmental colonic resection (n = 2) and gastric fundus resection (n = 1). The median (IQR) hospital stay was 12 (7.5) days. Vascular reconstruction was performed because of tumor involvement to the superior mesenteric vein in three patients, the superior mesenteric artery in two patients, and the splenic vein in one patient. Lymphovascular, perineural invasion, and lymph node metastasis were found in 5 (6.8%), 5 (6.8%), and 4 (5.4%) patients, respectively. According to the AJCC (TNM) Prognostic Groups, 9 (12%) patients were classified as stage I, 63 (85%) as stage II and, 2 (3%) as stage III.

**Table 2:** Surgical and pathological characteristics in 74 patients with solid pseudopapillary neoplasm who underwent surgery

	Total
	n^a^ = 74
Surgical procedures	
Pylorus-preserving pancreaticoduodenectomy, n (%)	30 (40.5)
Classic pancreaticoduodenectomy, n (%)	2 (2.7)
Distal pancreatectomy (with splenectomy), n (%)	26 (35.2)
Spleen-preserving distal pancretatecomy, n (%)	7 (9.4)
Central pancreatectomy, n (%)	9 (12.2)
Operative time,median (IQR)^b^, min	300 (168)
Estimated blood loss, median (IQR), mL	275 (275)
Vascular reconstruction, n (%)	6 (8.1)
Multivisceral resection, n (%)	9 (12.2)
Hospital stay, median (IQR), days	12 (7.5)
Resection margins status, n (%)	
R0	74(100)
R1	0
Lymphovascular invasion, n (%)	
Present	5(6.8)
Absent	60(81.1)
Perineural invasion, n (%)	
Present	5(6.8)
Absent	48(64.9)
Lymph nodes resected, median (IQR)	15(15)
Lymph node metastasis (pN), n (%)	4 (5.4)
AJCC^c^ (TNM)^d^ prognogtic stage group, n (%)	
I	9 (12)
II	63 (85)
III	2 (3)

^a^n: number of patients

^b^IQR: interquartile range

^c^AJCC: American joint committee on cancer

^d^TNM: tumor, nodes and metastases

### Postoperative outcomes

Postoperative outcomes were summarized in Table 3, Supplementary table 1 and Supplementary table 2, respectively. Postoperative complications developed in 40 (54%) patients. Pancreatic fistula was the most frequent complication (n = 29; 54%) followed by pancreatitis (n = 9; 17%), abdominal infectious fluid collection (n = 4; 7%) and PPH (n = 3; 6%). Reoperation was performed in five patients (6.8%) for hemorrhage (n = 3; 60%), intestinal obstruction (n = 1; 20%) and anastomotic leak (n = 1; 20%). The overall 90-day mortality was found in one (1.4%) patient who developed PPH.

**Table 3:** Postoperative outcomes

Characteristic	Value
Postoperative mortality within 90 days, n^a^ (%)	1(1.4%)
Postoperative complication within 90 days, n (%)	40 (54%)
Reoperation within 90 days, n (%)	5(6.8%)
Postoperative hemorrhage	3 (4%)
Intestinal Obstruction	1 (1.4%)
Anastomotic Leak	1 (1.4%)
Length of hospital stay, median (IQR)^b^, days	13.3(7.5)

^a^Number of patients

^b^IQR: interquartile range

### Recurrence, recurrence-free survival, and overall survival

**Fig. 2:**
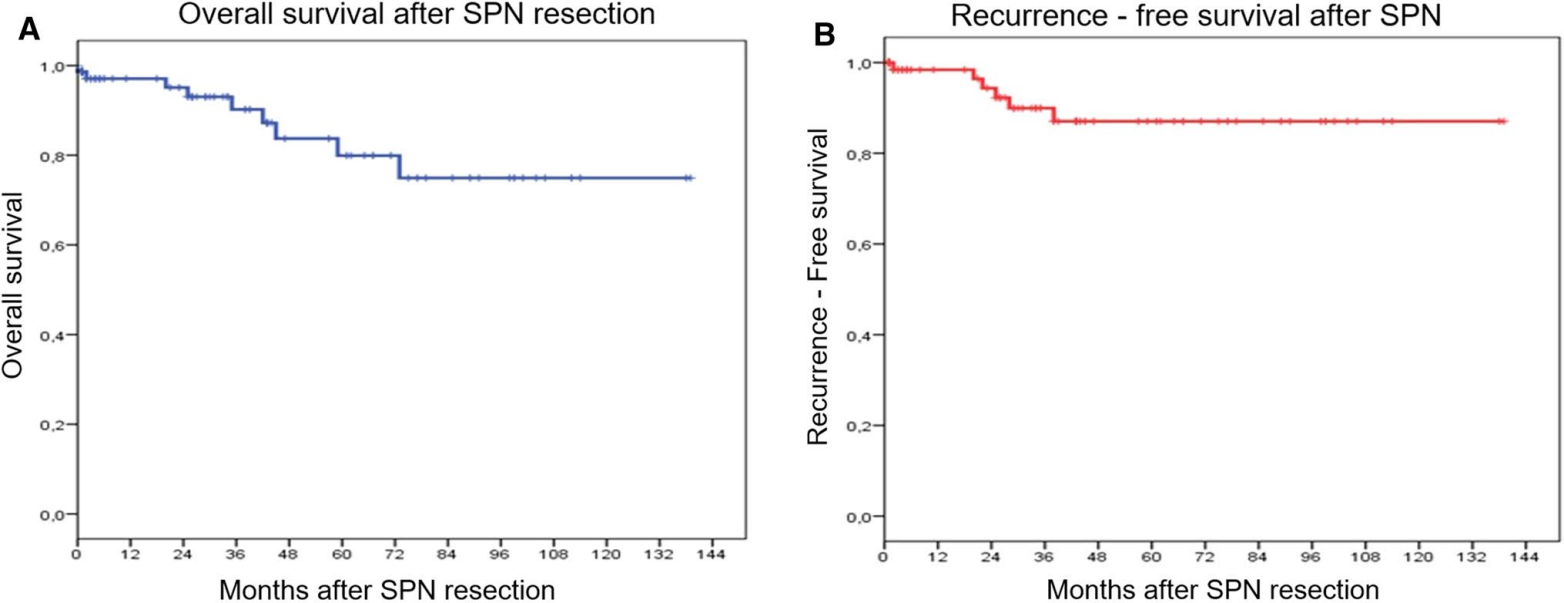
Overall survival (**A**) and reccurence-free survival (**B**) of patients with solid pseudopapillary neoplasm (SPN) who underwent pancreas resection

The median follow-up period was 40.2 (0.5–140) months. In our series, 6 (8%) patients developed recurrence after pancreas resection: liver metastasis (n = 5), peritoneal carcinomatosis (n = 2), and the remnant pancreas (n = 1); and 9 (12.2%) patients died. The 1-, 3-, and-5-year RFS was 98.4%, 89.9% and 87%, respectively. The 1-, 3-, and-5-year OS was 97.1%, 90.2% and 79.9%, respectively (Fig. 2). Demographic and pathologic characteristics of patients with recurrence were detailed in Table 4.

**Table 4:** Demographic and pathologic characteristics in 6 patients with solid pseudopapillary neoplasm who presented recurrence

Case	Age^a^	Sex	Tumor Location	Surgery	VR	MR	TD^b^	PNI	LVI	Margin	(pN)	Recurrence^c^	FollowUp ^d^	Treatment^e^	Status
1	38	F	Body	DP with en bloc splenectomy	No	Gastric Fundus	15	Absent	Present	R0	Negative	Liver	24	Chemotherapy: Gemcitabine + Cisplatin	Death
2	16	F	Head	Classic PD	SMV	No	11	NE	NE	R0	Positive	Liver	42	Chemotherapy: Gemcitabine + Cisplatin	Death
3	28	F	Head	Classic PD	No	Right Colectomy	20	Absent	Absent	R0	Negative	CarcinomatosisLiver	72	Palliative Treatment	Death
4	40	F	Tail	DP with en bloc splenectomy	No	No	14	NE	Absent	R0	Negative	Liver	20	Surgery	Death
5	51	F	Body	DP with en bloc splenectomy	No	No	6	Absent	Absent	R0	Negative	Pancreas Remanent	26	Surgery	Alive
6	40	F	Head	Pylorus-preserving PD	No	Right Colectomy	14	Absent	Absent	R0	Positive	CarcinomatosisLiver	20	Palliative Treatment	Death

VR, vascular reconstruction; MR, multivisceral resection; TD, tumor diameter; PNI, perineural invasion; LVI, lymphovascular invasion; pN, lymph node metastases; F, female; DP, distal pancreatectomy; PD, pancreaticoduodenectomy; SMV, superior mesenteric vein; R0, no microscopic residual tumor; NE, not evaluated

^a^At the date of surgery

^b^Tumor diameter in centimeters

^c^Recurrence site

^d^Follow up in months after surgery

^e^Treatment of disease recurrence

### Cox proportional hazards model analysis for RFS

**Fig. 3:**
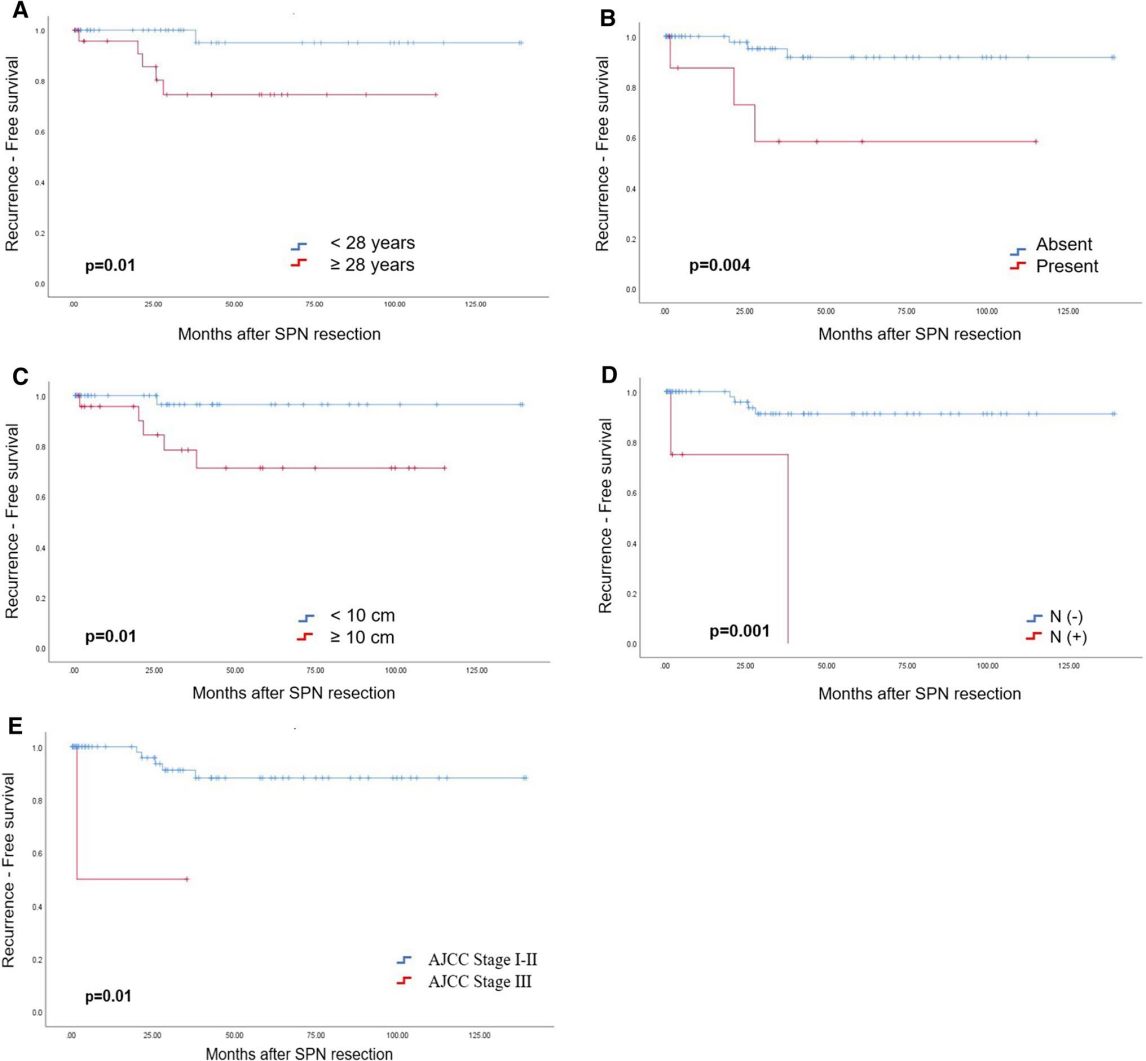
Kaplan–Meier curves of recurrence-free survival in patients with SPN comparing: **A** patients < 28 years vs ≥ 28 years old; **B** Invasion of adjacent organs absent vs present; **C** Tumor diameter < 10 cm vs ≥ 10 cm; **D** Lymph node metástasis N (+) vs N (−); **E** AJCC Stage I-II vs AJCC stage III

In a univariable Cox proportional hazard model analysis, age ≥ 28 years (HR = 8.61, 95% CI 1.1–73.8), tumor diameter ≥ 10 cm (HR = 9.3, 95% CI 1.12–79.6), invasion of adjacent organs (HR = 7.45, 95% CI 1.5–36.9), lymph node metastasis (N +) (HR = 16.8, 95% CI 2.96–94.9) and, AJCC (TNM) Stage III (HR = 10.1, 95% CI 1.2–90.9) were associated with risk factors for recurrence in patients (Table 5) (Fig. 3).

**Table 5:** Univariate Cox regression analysis of RFS

Factor	No. of patients	No. of events	5-year RFS (%)	Univariable analysis		
				HR	95% CI	p-value
Age^a^, years						
< 28	49	1	98.0	Reference		
≥ 28	25	5	80.0	8.61	(1.1–73.8)	0.04
Tumor diameter^b^						
< 10 cm	50	1	98.0	Reference		
≥ 10 cm	24	5	79.0	9.3	(1.12–79.6)	0.042
Invasion to adjacent organs						
No	65	3	91.8	Reference		
Yes	9	3	58.3	7.45	(1.5–36.9)	0.014
Lymph node metastasis (pN)						
Absent	70	4	91.0	Reference		
Present	4	2	0.0	16.8	(2.96–94.9)	0.001
AJCC stage						
I–II	72	5	93.0	Reference		
III	2	1	50.0	10.1	(1.2–90.9)	0.03

RFS, recurrence-free survival; HR, Hazard ratio; CI, confidence interval; AJCC, American joint committee on cancer

^a^At the date of surgery

^b^Tumor diameter in centimeters

## Discussion

SPN is a relatively rare disease and is generally regarded as a low malignant potential [27]. In recent years, the number of publications on this topic has been increasing but the risk factors associated with recurrence remain unclear. This may be related to the small number of cases reported worldwide and the difficulty of having a large single-center series [6]. Our study presented the clinicopathological characteristics of SPN patients in Peru and contributed to clarifying the risk factor for recurrence after surgical resection. In the present study, it was shown that age ≥ 28 years old, tumor diameter ≥ 10 cm, invasion of adjacent organs, lymph node metastasis, and AJCC stage III were associated with recurrence in SPN patients.

Several studies reported that age at diagnosis was related to the prognosis of patients with resected SPN [28, 29]. Waters et al. [28] reported in a retrospective study based on the United States National Cancer Database that pediatric patients (≤ 21 years) were significantly associated with better overall survival than adult patients (≥ 22 years). In contrast, in a retrospective study conducted in France, it was reported that patients under 13.5 years old were significantly associated with worse disease-free survival compared to patients ≥ 13.5 years old [29]. According to our results, younger patients (< 28 years old) presented a better prognosis compared to older patients (≥ 28 years old). These results are in line with previous studies reporting similar results and lead to a new hypothesis about biological differences between these groups of patients and their future significance in predicting the possibility of disease recurrence.

A larger tumor diameter has been reported as a risk factor for recurrence in SPN patients in previous studies [15, 19]. Gao et al. [19], in a systematic review, reported that SPN patients with a tumor diameter > 5 cm presented a higher risk of recurrence compared to patients with a tumor diameter ≤ 5 cm (OR = 4.74, 95% CI 1.12–20.05). Likewise, a retrospective multicenter study conducted in Korea showed in a multivariate analysis, that a tumor diameter > 8 cm was significantly associated with recurrence in resected SPN patients [15]. In the present study, SPN patients with a tumor diameter ≥ 10 cm presented a higher risk of recurrence in the Cox proportional hazard model analysis (HR = 9.3, 95% CI 1.12–79.6). This result could be attributable to the relatively larger tumor diameter in our study compared to the tumor diameter in previous studies [4, 14, 15, 30]. Also, our study enhances a new cut-off value (tumor diameter  ≥ 10 cm) to predict the possibility of recurrence in resected SPN patients and introduce a new line of research regarding the association between the tumor diameter and recurrence in SPN.

Nine (12.2%) patients presented invasion of adjacent organs and required a multivisceral resection to ensure free surgical margins (R0), of these, three patients presented recurrence. In the Cox proportional hazard model analysis, the presence of invasion of adjacent organs was significantly related to recurrence in resected SPN patients (HR = 7.45, 95% CI 1.5–36.9). Likewise, several studies also reported that the invasion of the peripancreatic fat tissue and adjacent organs was related to a worse prognosis in SPN patients [31, 32]. These results support the importance of a complete oncological surgical resection (R0) in patients with the diagnosis of SPN because of his malignant potential. In addition, we suggest that if an adjacent organ is compromised by the primary tumor, it must be resected en-bloc to ensure a better prognosis.

The role of performing a routine lymphadenectomy in SPN patients is currently debatable [33]. Lymphatic involvement in patients with the diagnosis of SPN has been reported in several studies with a percentage up to 8% [4, 29, 34]. Gao et al. [19], reported that the presence of positive lymph nodes metastasis was significantly related to postoperative relapse in patients with resected SPN (OR: 6.58, 95 CI 1.92–22.57). In the present study, four (5.4%) patients presented lymph node metastasis, and two of them presented recurrence. Similarly, in the Cox proportional hazard model analysis, the presence of lymph node metastasis was significantly associated with recurrence (HR: 16.8, CI 95% 2.96–94.9). According to our results, we proposed that surgical resection of the primary tumor should be complemented with a lymphadenectomy to ensure better staging in SPN patients.

In the present study, patients were categorized according to the AJCC (TNM) Prognostic Group classification. Patients who were categorized as Stage III presented a higher risk of recurrence compared to the patients categorized as Stage I and II. In a retrospective study conducted at the MD Anderson Cancer Center, similar results were reported, in this study, the AJCC staging group classification was significantly associated with recurrence (p ≤ 0.01) [4]. According to these results, the AJCC classification should be used systematically to categorize patients with resected SPN, to perform it, we should also have several resected lymph nodes to assess the lymph node involvement and subsequently evaluate the long-term prognosis.

In previous studies, the recurrence rates in resected SPN are relatively low, ranging from 1.5 to 13.7% and the liver was the most frequent recurrence site. In the current study, 6 (8%) patients developed recurrence including liver metastases and peritoneal carcinomatosis. In our institution, SPN patients are treated with complete oncological resection of the primary tumor and multivisceral resection in case of involvement of adjacent organs. Likewise, lymphadenectomy is performed in all cases for treatment and staging. Our results are comparable with other studies worldwide and even with a lower recurrence rate.

In our series, 12.6% of all pancreatic resections were histologically diagnosed as SPN. In Peru, the National Institute of Neoplastic Diseases is the national reference center for oncological pancreatic surgeries especially in this pathology, this could explain the high percentage of SPN in our institution. However, more studies are required to analyze whether there is a higher incidence of SPN in the Latin American population.

In the present study, most patients were female (94.6%) and only four patients were male. This is in line with previous studies which showed that SPN was predominant in females suggesting a hormonal factor [35, 36]. Also, studies reported that male patients with SPN present an incidence at a higher age and a compromised survival after surgery [14, 37]. In our series, the oldest patient was a 68-year-old male; nonetheless, no recurrence was found in male patients. SPN is generally found with symptoms [29, 38, 39]. In our series, 70 (94.6%) patients had symptoms with the most frequent symptom, abdominal pain. Currently, there is no evidence of a prognostic role of tumor markers in patients with SPN [3, 40, 41]. In our study, Ca 19-9 levels were higher in patients with recurrence compared to patients without recurrence, but no significant difference was observed (p = 0.21). The pancreas tail is the most common location of SPN [34, 38, 42, 43]. However, our study showed that the pancreas head was the most frequent location of SPN (n = 32; 43.2%) followed by the pancreas tail (26; 35.2%). This is similar to a report in the Chinese population, which showed that 39.8% of SPN were located at the pancreas head in 553 patients [44].

Our study should be understood in the context of limitations. First, our study is conducted under the retrospective study design with a relatively small number of patients. Nonetheless, the diagnosis of SPN is rare, and case series are scarce worldwide. Our study is the largest series in Latin America. The functional change after pancreatectomy was not followed because most patients live in remote areas far from our institution.

## Conclusions

In conclusion, SPN is frequently diagnosed in young females. The prognosis is good after R0 resection. However, recurrence may develop in some patients. Our study found that age ≥ 28 years, tumors ≥ 10 cm, invasion to adjacent organs, lymph node metastasis and, AJCC Stage III were risk factors of recurrence in patients undergoing SPN resection.

## Supplementary Information

Additional file 1 (XLSX 13 KB)

Additional file 2 (XLSX 12 KB)

## Data Availability

The datasets used and/or analyzed during the current study are available from the corresponding author on reasonable request.

## References

[1] Frantz V (1959). Tumors of the pancreas. Atlas of tumor pathology.

[2] Yu P, Cheng X, Du Y (2015). Solid pseudopapillary neoplasms of the pancreas: a 19-year multicenter experience in China. J Gastrointest Surg.

[3] Xu Y, Zhao G, Pu N (2017). One hundred twenty-one resected solid pseudopapillary tumors of the pancreas: an 8-year single-institution experience at Zhongshan Hospital, Shanghai, China. Pancreas.

[4] Estrella JS, Li L, Rashid A (2014). Solid pseudopapillary neoplasm of the pancreas: clinicopathologic and survival analyses of 64 cases from a single institution. Am J Surg Pathol.

[5] Law JK, Ahmed A, Singh VK (2014). A systematic review of solid-pseudopapillary neoplasms: are these rare lesions?. Pancreas.

[6] Lanke G, Ali FS, Lee JH (2018). Clinical update on the management of pseudopapillary tumor of pancreas. World J Gastrointest Endosc.

[7] Tanaka Y, Kato K, Notohara K (2001). Frequent beta-catenin mutation and cytoplasmic/nuclear accumulation in pancreatic solid-pseudopapillary neoplasm. Cancer Res.

[8] Tiemann K, Kosmahl M, Ohlendorf J (2006). Solid pseudopapillary neoplasms of the pancreas are associated with FLI-1 expression, but not with EWS/FLI-1 translocation. Mod Pathol.

[9] Bergmann F, Andrulis M, Hartwig W (2011). Discovered on gastrointestinal stromal tumor 1 (DOG1) is expressed in pancreatic centroacinar cells and in solid-pseudopapillary neoplasms—novel evidence for a histogenetic relationship. Hum Pathol.

[10] Adachi S, Jigami T, Yasui T (2004). Role of a BCL9-related beta-catenin-binding protein, B9L, in tumorigenesis induced by aberrant activation of Wnt signaling. Cancer Res.

[11] Zalatnai A, Kis-Orha V (2020). Solid-pseudopapillary neoplasms of the pancreas is still an enigma: a clinicopathological review. Pathol Oncol Res.

[12] Reddy S, Cameron JL, Scudiere J (2009). Surgical management of solid-pseudopapillary neoplasms of the pancreas (Franz or Hamoudi tumors): a large single-institutional series. J Am Coll Surg.

[13] World Health Organization Classification of Tumours (2010). WHO classification of tumours of the digestive system.

[14] Machado MC, Machado MA, Bacchella T (2008). Solid pseudopapillary neoplasm of the pancreas: distinct patterns of onset, diagnosis, and prognosis for male versus female patients. Surgery.

[15] Kang CM, Choi SH, Kim SC (2014). Predicting recurrence of pancreatic solid pseudopapillary tumors after surgical resection: a multicenter analysis in Korea. Ann Surg.

[16] Papavramidis T, Papavramidis S (2005). Solid pseudopapillary tumors of the pancreas: review of 718 patients reported in English literature. J Am Coll Surg.

[17] Matos JM, Grützmann R, Agaram NP (2009). Solid pseudopapillary neoplasms of the pancreas: a multi-institutional study of 21 patients. J Surg Res.

[18] Wright MJ, Javed AA, Saunders T (2020). Surgical resection of 78 pancreatic solid pseudopapillary tumors: a 30-year single institutional experience. J Gastrointest Surg.

[19] Gao H, Gao Y, Yin L (2018). Risk factors of the recurrences of pancreatic solid pseudopapillary tumors: a systematic review and meta-analysis. J Cancer.

[20] Klóppel G, Basturk O, Klimstra D, Lam AK, Notohara K, WHO Classification of Tumours Editorial Board (2019). Solid pseudopapillary neoplasm of the pancreas. Digestive system tumours.

[21] Ferrone CR, Konstantinidis IT, Sahani DV (2011). Twenty-three years of the Warshaw operation for distal pancreatectomy with preservation of the spleen. Ann Surg.

[22] Kimura W, Yano M, Sugawara S (2007). Spleen-preserving distal pancreatectomy with conservation of the splenic artery and vein: techniques and its significance. J Hepatobiliary Pancreat Sci.

[23] Dindo D, Demartines N, Clavien PA (2004). Classification of surgical complications: a new proposal with evaluation in a cohortof 6336 patients and results of a survey. Ann Surg.

[24] Bassi C, Dervenis C, Butturini G (2005). Postoperative pancreatic fistula: an international study group (ISGPF) definition. Surgery.

[25] Bassi C, Marchegiani G, Dervenis C (2017). The 2016 update of the international study group (ISGPS) definition and grading of postoperative pancreatic fistula : 11 years after. Surgery.

[26] Wente MN, Veit JA, Bassi C (2007). Postpancreatectomy hemorrhage (PPH): an International study group of pancreatic surgery (ISGPS) definition. Surgery.

[27] Bhutani N, Kajal P, Singla S (2017). Solid pseudopapillary tumor of the pancreas: experience at a tertiary care centre of Northern India. Int J Surg Case Rep.

[28] Waters AM, Russell RT, Maizlin II (2019). Comparison of pediatric and adult solid pseudopapillary neoplasms of the pancreas. J Surg Res.

[29] Irtan S, Galmiche-Rolland L, Elie C (2016). Recurrence of solid pseudopapillary neoplasms of the pancreas: results of a nationwide study of risk factors and treatment modalities. Pediatr Blood Cancer.

[30] Marchegiani G, Crippa S, Malleo G (2011). Surgical treatment of pancreatic tumors in childhood and adolescence: uncommon neoplasms with favorable outcome. Pancreatology.

[31] Serrano PE, Serra S, Al-Ali H (2014). Risk factors associated with recurrence in patients with solid pseudopapillary tumors of the pancreas. JOP.

[32] Kim CW, Han DJ, Kim J (2011). Solid pseudopapillary tumor of the pancreas: can malignancy be predicted?. Surgery.

[33] Son J, Kim W, Seo JM (2021). Prediction of recurrence of completely resected pancreatic solid pseudopapillary neoplasms in pediatric patients: a single center analysis. Children.

[34] Huffman BM, Westin G, Alsidawi S (2018). Survival and prognostic factors in patients with solid pseudopapillary neoplasms of the pancreas. Pancreas.

[35] Cai YQ, Xie SM, Ran X (2014). Solid pseudopapillary tumor of the pancreas in male patients: report of 16 cases. World J Gastroenterol.

[36] Milanetto AC, Gais Zürcher AL, Macchi L (2020). Pancreatic solid pseudopapillary neoplasm in male patients: systematic review with three new cases. Updates Surg.

[37] Kuo EJ, Salem RR (2015). Men in the US with solid pseudopapillary carcinomas of the pancreas have compromised survival: a population-level study of outcomes. J Pancreas.

[38] Butte JM, Brennan MF, Gönen M (2011). Solid pseudopapillary tumors of the pancreas. Clinical features, surgical outcomes, and long-term survival in 45 consecutive patients from a single center. J Gastrointest Surg..

[39] Hwang J, Kim DY, Kim SC (2014). Solid-pseudopapillary neoplasm of the pancreas in children: can we predict malignancy?. J Pediatr Surg.

[40] El Nakeeb A, Abdel Wahab M, Elkashef WF (2013). Solid pseudopapillary tumour of the pancreas: Incidence, prognosis and outcome of surgery (single center experience). Int J Surg.

[41] Lee S, Jang JY, Hwang DW (2008). Clinical features and outcome of solid pseudopapillary neoplasm differences between adults and children. Arch Surg.

[42] Yang F, Fu DL, Jin C (2008). Clinical experiences of solid pseudopapillary tumors of the pancreas in China. J Gastroenterol Hepatol.

[43] Marchegiani G, Andrianello S, Massignani M (2016). Solid pseudopapillary tumors of the pancreas: specific pathological features predict the likelihood of postoperative recurrence. J Surg Oncol.

[44] Yu PF, Hu ZA, Wang XB (2010). Solid pseudopapillary tumor of the pancreas: a review of 553 cases in Chinese literature. World J Gastroenterol.

